# Stress reduction through taiji: a systematic review and meta-analysis

**DOI:** 10.1186/s12906-024-04493-3

**Published:** 2024-06-03

**Authors:** Jana Kraft, Paula J. Waibl, Karin Meissner

**Affiliations:** 1 Department of Applied Natural Sciences and Health, Programs in Health Promotion, Coburg University of Applied Sciences, Friedrich-Streib-Str. 2, Coburg, 96450 Germany; 2 Institute of Medical Psychology, Medical Faculty, LMU Munich, Munich, Germany

**Keywords:** Mind–body exercise, Tai Chi, Stress reduction, Relaxation, Evidence-based practice

## Abstract

**Purpose:**

In light of the mounting prevalence of stress in contemporary society and the growing interest in stress reduction methods, this review investigates the potential of taiji as a viable strategy for alleviating stress.

**Methods:**

MEDLINE, EMBASE, the Cochrane Controlled Trials Register (CENTRAL), PsycINFO, and Web of Science were searched up to April 2023 to identify randomized controlled trials of taiji. Studies in both patients and healthy populations were considered. They had to provide a measure of perceived stress and include a no treatment or placebo control group. Data were extracted by two reviewers. Pooled standardized mean differences (SMD) were calculated for perceived stress, biological stress markers, anxiety, depression, and quality of life (QoL). Meta-regression analyses were performed to identify sources of heterogeneity.

**Results:**

Eleven trials with a total of 1323 patients comparing taiji to no intervention met the inclusion criteria. The included studies varied strongly with regard to patient characteristics, taiji intervention, and methodological quality. The overall SMD for perceived stress was significant at -0.41 (95% confidence interval, CI, -0.63 to -0.19; I^2^ = 63%). Exclusion of studies with less than 100 participants yielded a diminished SMD at -0.26 (95% CI, -0.45 to -0.06). The SMD for perceived stress at follow-up was significant (-0.25, 95% CI -0.46 to -0.05). Secondary outcomes highlighted improvements in anxiety and physical QoL, while depression, mental QoL, and biological stress markers remained unchanged.

**Conclusions:**

Results underscore taiji's potential in mitigating perceived stress in both patients and healthy populations, paralleled by enhancements in depressive symptoms, anxiety levels, and physical QoL.

## Introduction

Stress, a ubiquitous component of modern life, has a multifaceted impact on health. It can exert direct effects through autonomic and neuroendocrine pathways or indirectly influence health behaviors [1]. While acute stress can enhance cognitive and physical performance, chronic and intense stress contributes to a variety of health issues, ranging from obesity and hypertension to migraine, asthma, and depression [2].

In light of the pervasive nature of stress in contemporary societies, stress reduction techniques have become quite popular. One influential mind–body technique rooted in Eastern traditions is taiji (also known as tai chi). Originally developed as a martial art, taiji is a form of mindful moving technique, combining slow and flowing movements with mindful breathing [3–5]. Over recent years, scientific interest in the health-enhancing potential of taiji has increased, and beneficial effects on physiological and psychological well-being have been confirmed [3, 6].

Several systematic reviews and meta-analyses on taiji included outcomes related to stress and psychological well-being. For example, in adolescents and college students, there is increasing evidence that taiji can improve depression, anxiety, stress, and cortisol levels [1, 2]. Beneficial effects of taiji in mitigating work-related stress among health professionals have also been reported [3]. A meta-analysis in patients with cardiovascular disease demonstrated favorable effects of taiji for anxiety, depression, and quality of life (QoL) [4, 5]. In cancer survivors, taiji positively impacted fatigue and sleep quality, while there were no significant improvements in anxiety, stress, depressive symptoms, or overall QoL [6].

Aside from partly inconsistent results, these findings are limited by small numbers of participants [7] and the partly low quality of included studies [1, 3, 4]. For example, many of these studies do not adhere to current methodological standards, including the incorporation of a control group or randomized allocation to treatment and control groups [8]. Hence, more comprehensive reviews and meta-analyses are needed to conclusively determine the stress-relieving potential of taiji.

This review aimed to systematically evaluate the available evidence from randomized controlled trials that taiji reduces perceived stress. Secondary outcomes encompassed physiological stress indicators, depression, anxiety, and health-related QoL. Our evaluation considered both patients and healthy populations.

## Methods

This systematic review was conducted and reported according to the Preferred Reporting Items for Systematic Reviews and Meta-Analyses (PRISMA) standard [9].

### Literature search

The literature search was performed by two reviewers (KM, JK) as part of a larger systematic review evaluating the stress-reducing potential of various yangsheng techniques, namely taiji, qigong, and acupressure. The following databases were searched: MEDLINE, EMBASE, the Cochrane Controlled Trials Register (CENTRAL), PsychINFO, and Web of Science (from inception to April 2023) using a combination of keywords and text words related to stress, relaxation, and taiji, qigong, and acupressure, combined with validated filters for controlled clinical trials. The search strategy for MEDLINE is shown in Table 1. Further potentially relevant publications were retrieved directly from the references cited in RCTs and systematic reviews.

**Table 1:** Search strategy for MEDLINE

No	Search term
# 1	(randomised controlled trial or randomized controlled trial).pt
# 2	placebo.ab
# 3	drug therapy.fs
# 4	random*.ab
# 5	trial.ab
# 6	groups.ab
# 7	1 or 2 or 3 or 4 or 5 or 6
# 8	exp animals/ not humans.sh
# 9	7 not 8
# 10	psychological stress.mp. or exp stress, psychological/
# 11	exp relaxation/ or relaxation.mp. or exp muscle relaxation/ or exp relaxation therapy/
# 12	10 or 11
# 13	cortisol.mp. or exp hydrocortisone/ or heart rate varability.mp. or exp norepinephrine/ or norepinephrine.mp. or epinephrine.mp. or exp epinephrine/ or adrenaline.mp. or noradrenaline.mp. or alpha-amylase.mp. or exp alpha-Amylases/ or exp Heart Rate/
# 14	12 and 13
# 15	12 or 14
# 16	qigong.mp. or exp qigong/ or Qi gong.mp. or ch’i kung.mp. or chi kung.mp. or baduanjin.mp
# 17	tai ji.mp. or exp tai ji/ or tai chi.mp. or tai-ji.mp. or taiji.mp. or taijiquan.mp
# 18	acupressure.mp. or exp acupressure/
# 19	16 or 17 or 18
# 20	9 and 15 and 19

### Eligibility criteria

Only randomized controlled trials were considered. Study protocols were excluded. Participants could come from any demographic, including patients, healthy volunteers, or specific occupational groups. The primary intervention must have been taiji. Studies combining taiji with other interventions, such as relaxation methods or psychotherapy, were excluded. Interventions needed to last at least four weeks. Control groups were required to receive either a placebo treatment, or treatment as usual, or no intervention, such as being placed on a waiting list (WL) or receiving no treatment (NT). Eligible studies needed to provide a measure of perceived stress.

The primary outcome was the level of perceived stress at the conclusion of the intervention in the taiji group compared to the control group (placebo, WL, or NT). Stress had to be measured using a validated questionnaire or, alternatively, through visual analogue scales or numeric analogue scales. Secondary outcomes encompassed perceived stress at the latest available follow-up, stress compared to additional active control groups (typically those engaging in physical activity), as well as biological stress markers such as systolic blood pressure (BP), diastolic BP, heart rate, and cortisol levels. Additional secondary outcomes included psychological distress indicators like depression and anxiety, as well as both mental and physical QoL at the intervention's end.

### Study selection

All abstracts identified through the literature search were screened, and irrelevant hits, such as nonrandomized studies and study protocols, were excluded. All remaining articles were obtained in full text and checked for eligibility based on the predefined selection criteria.

### Data collection

Data was extracted using a standardized Excel form that captured the following details: bibliographic study information; study population demographics, including the age and gender of participants; numbers of participants involved at each stage (randomization, intervention, and analysis) as well as dropouts; details of the taiji intervention (type, duration, frequency) and, where relevant, sham interventions; nature of the control group; duration of both the intervention and follow-up periods; study design specifics (e.g., parallel-group or crossover); predefinition of primary outcomes; and type of analysis (intent-to-treat or per protocol). The data extraction for each study was independently performed by two of the three reviewers (KM, JK, PW), and any disagreements were resolved through discussion.

### Risk of bias assessment

The risk of bias was assessed using the Cochrane Collaboration's risk of bias tool [10]. For each of the following domains – random sequence generation, allocation concealment, blinding of participants and personnel, blinding of outcome assessors, incomplete outcome data, and selective reporting – the risk of bias was categorized as high, low, or unclear. The risk of bias assessment was performed for each study by two of the three reviewers (KM, JK, PW), and any disagreements were clarified through discussion. Summary graphs depicting the risk of bias were generated using Review Manager (RevMan) version 5.3..

### Statistical analyses

Meta-analyses were conducted using RevMan. For the meta-analyses of the primary outcomes, standardized mean differences (SMD) between the intervention group and the control group and their 95% confidence intervals (CI) were calculated for each study. Results were then pooled using random-effects models. To check the robustness of results, sensitivity analyses were performed for (1) studies with low risk of bias related to allocation concealment, blinding of outcome assessors, and incomplete outcome data; (2) studies with total sample sizes of ≥ 100; and (3) studies with clearly predefined primary outcome measures (≤ 2 pre-specified primary outcomes, including how and when they were assessed). The effects of study characteristics on primary outcomes were investigated using multivariable meta-regression analyses by in IBM SPSS Statistics (version 25). For the meta-analyses of the secondary outcomes, standardized mean difference (SMD) between the intervention group and the respective control group and their 95% confidence intervals (CI) were calculated for each study.

Statistical heterogeneity was examined using Cochrane’s Q test and the I^2^ statistics. I^2^ values of 25%, 50%, and 75% were considered as low, moderate, and high heterogeneity, respectively. Funnel plot asymmetry was assessed using Egger’s test as a measure of publication bias [11]. The Egger’s test was performed using SPSS. For all statistical tests, a *p*-value < 0.05 was considered significant.

## Results

### Literature search

**Fig. 1:**
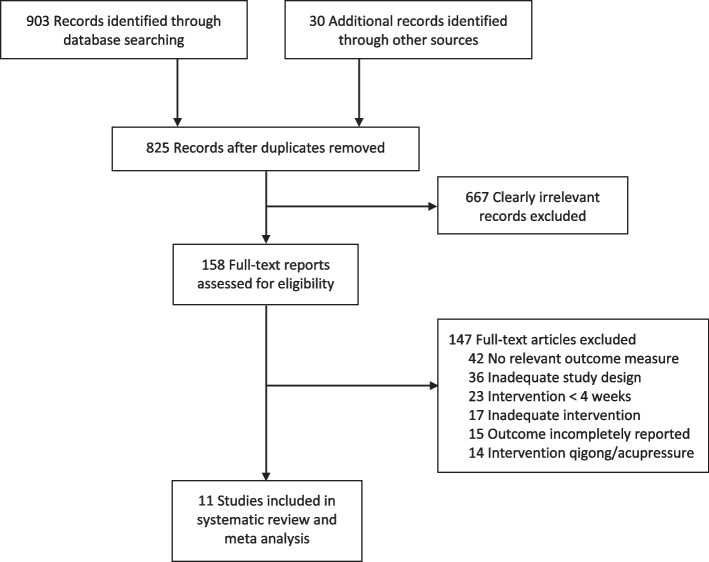
Flow diagram

The study selection process is shown in Fig. 1 [12]. The literature search yielded 933 hits, of which 108 were duplicates. Upon screening the abstracts, 667 clearly irrelevant records were excluded. The full texts of 158 potentially relevant hits were retrieved, from which 133 full text articles were excluded because they did not meet the inclusion criteria or examined qigong or acupressure interventions. Eleven RCTs on taiji were included in this review.

### Characteristics of included studies

Table 2 provides a summary of the characteristics of the eleven included studies [13–23]. Most of the studies were published in the last ten years. In total, 1323 participants were randomly assigned to either an experimental taiji intervention, or NT (7 trials WL, 3 trials NT, 1 trial TAU), or an additional active control group (5 trials, with different types of exercise). Five trials investigated patients and six trials healthy/stressed populations. The median age of participants was 53 years (IQR, 34–64), with men comprising 29% (median; IQR, 16–36) of the participants. Four of the trials had a clearly predefined primary outcome measure, four trials a partly predefined primary outcome measure (like predefinition of the outcome but not the time point, or more than two outcomes predefined as primary), and three trials had no predefined primary outcome measure. The duration of taiji practice ranged from 45 to 120 min, one to five times weekly, spanning 8 to 24 weeks (not counting self-practice at home). Table 3 gives an overview of the applied taiji interventions.

**Table 2:** Characteristics of included studies

Study ID	Country	Participants	Intervention 1 (Intervention 2)	Control	N rando-mized	Duration of intervention, weeks	Mean age, years	Stress (S) outcome	Anxiety (A)/ Depression (D) outcome	QOL outcome
**Chan 2018 ** [23]	Hong Kong	Adults with hypertension	taiji (Brisk walking)	NT	246	12	64	PSS SBP, DBP	n/a	SF-12 (physical, mental)
**Fransen 2007 ** [22]	Australia	Patients with chronic symptomatic hip or knee osteoarthritis	taiji (Hydrotherapy)	WL	152	12	70	DASS-S	DASS-D, DASS-A	SF-12 (physical, mental)
**Ho 2016 ** [21]	Hong Kong	Patients with chronic schizophrenia	taiji (Control exercise)	WL	153	12	54	PSS, cortisol	PANSS-D	n/a
**Lauche 2016 ** [20]	Germany	Patients with chronic nonspecific neck pain	taiji (Neck exercise group)	WL	114	12	49	PSS	HADS-A, HADS-D	SF-36 (physical, mental)
**Liu 2015 ** [19]	Australia	Obese adults with depression symptoms	taiji	TAU	213	24	53	DASS-S, SBP, DBP	DASS-D, DASS-A	n/a
**Nedeljkovic 2013 ** [18]	Switzerland	Healthy participants	taiji	WL	70	12	35	PSS	n/a	n/a
**Palumbo 2012 ** [17]	USA	Older nurses	taiji	NT	14	15	54	PSS	n/a	SF-36 (mental)
**Solianik 2021 ** [16]	Lithuania	Healthy but physically inactive adults aged ≥ 60 years	taiji	NT	30	10	67	PSS, SBP, DBP, HR	HADS-A HADS-D	n/a
**Zhang 2018 ** [15]	China	Adolescents with subthreshold depression	taiji	WL	64	8	18	PSS	PHQ-9 (D)	n/a
**Zheng 2015 ** [14]	China	College students	taiji	WL	198	12	21	PSS, SBP, DBP, HR	n/a	WHOQOL-BREF (medians only)
**Zheng 2018 ** [13]	Australia	Stressed but healthy people	taiji (Exercise)	WL	69	12	34	PSS, SBP, DBP, HR Variability	STAI-Trait	SF-36 (mental)

*Abbreviation*s: *DASS* Depression anxiety stress scale, *DBP* Diastolic blood pressure, *HADS* Hospital anxiety and depression scale, *HR* Heart rate, *n/a* Not applicable, *NT* No treatment, *PANSS* Positive and negative syndrome scale, *PHQ* Patient health questionnaire, *PSS* Perceived stress scale, *SF* Short form health survey, *STAI* State-trait-anxiety inventory, *SBP* Systolic blood pressure, *TAU* Treatment as usual, *WHOQOL-BREF* WHO Quality of Life-BREF, *WL* Waiting list control

**Table 3:** Main characteristics of taiji treatments

Study ID	Taiji style	Treatment frequency	Treatment aim
Chan 2018 [23]	24-form Yang Style taiji	60 min group sessions twice weekly for 3 months; and 30 min home practice per day on ≥ 5 days per week	To investigate the effects of taiji on cardiovascular risk factors and psychosocial well-being in adults with hypertension
Fransen 2007 [22]	24-form Sun Style taiji	60 min group sessions twice weekly for 12 weeks, and home practice (not monitored)	To investigate the clinical benefits of taiji for individuals with chronic symptomatic hip or knee osteoarthritis
Ho 2016 [21]	Chen-form Wu-style taiji (1st°segment)	60 min group sessions each week for 3 months; and twice-weekly a 45-min practice session	To elucidate the benefits of taiji, such as attainment of mental tranquility and relaxation, in patients with chronic schizophrenia
Lauche 2016 [20]	Yang style taiji (13 forms from Mantak Chia)	75–90-min group session once weekly for 12 weeks	To test the efficacy of taiji for treating chronic neck pain
Liu 2015 [19]	Adapted Kaimai style taiji	60–90 min group sessions 3 times per week for 24 weeks; and home practice 4 times per week	To examine the effects of taiji on depression, anxiety, and stress in centrally obese people with elevated depression symptoms
Nedeljkovic 2013 [18]	First 18 sequences of 37°Chen Man-Ch’ing Yang-Style taiji, short form	60 min group sessions twice per week for 12 weeks	To examine the effects of taiji on perceived stress and general self-efficacy in healthy participants
Palumbo 2012 [17]	Simplified Yang style taiji	45-min group session once a week for 15 weeks; and 10-min home-practice on ≥ 4 days per week	To improve health and well-being, physical functions, work limitations, stress, and work productivity in older nurses
Solianik 2021 [16]	8-form Yang Style taiji	60 min group sessions twice per week for 10 weeks	To determine the effects of taiji on psychoemotional state, cognition, and motor learning in physically inactive adults (≥ 60 y.)
Zhang 2018 [15]	Simplified 24°short-form taiji combined with mindfulness skills	90 min group sessions twice weekly for 8 weeks	To evaluate the effect of taiji on depression and mindfulness in adolescents with subthreshold depression
Zheng 2015 [14]	Simplified 24°forms of taiji	60 min group sessions, five days per week for 12 weeks	To investigate the effectiveness and safety of taiji on physical and psychological health of college students
Zheng 2018 [13]	Simplified 24°forms of taiji	2 h of supervised taiji per week for 6 weeks, followed by home practice for 6 weeks	To determine whether 12 weeks of taiji reduce anxiety in healthy but stressed people

### Risk of bias assessment

**Fig. 2:**
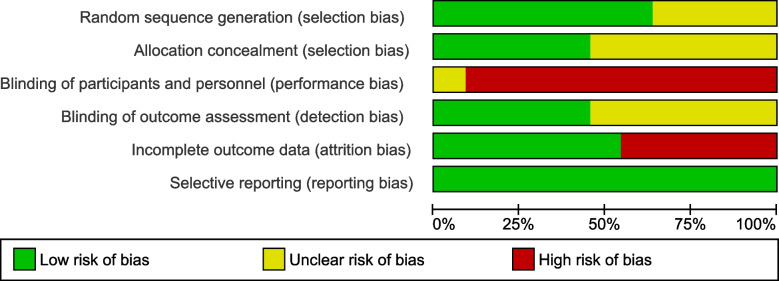
Risk of bias graph

**Fig. 3:**
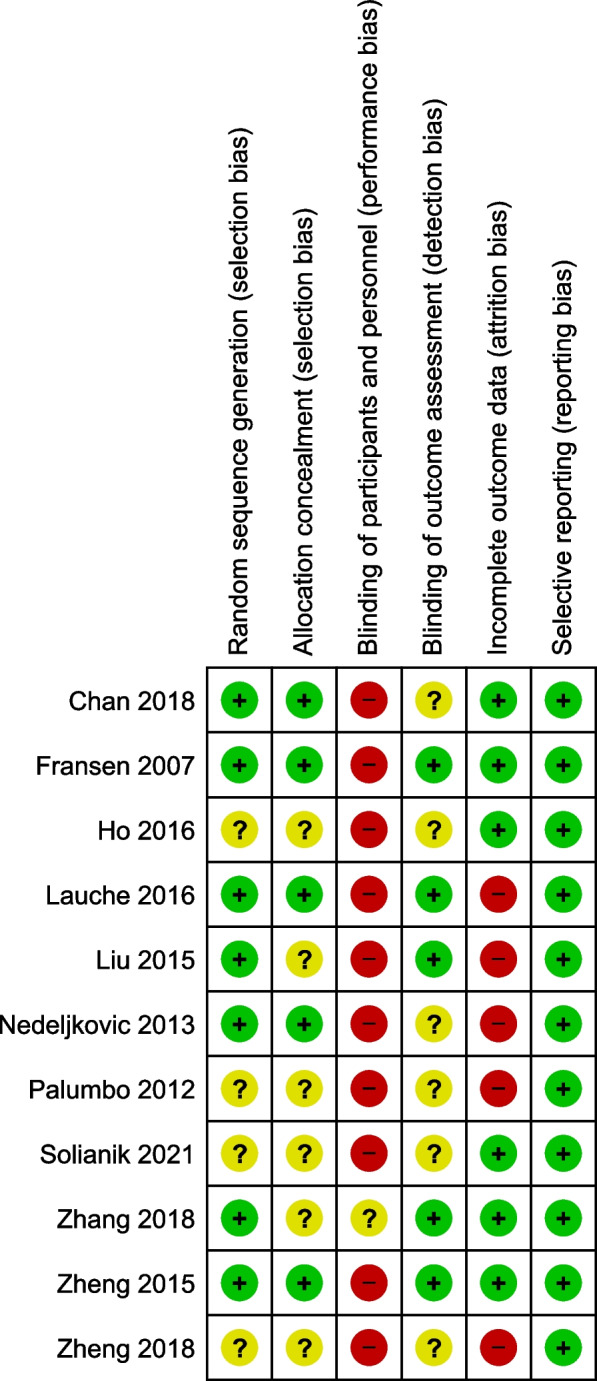
Risk of bias summary

About half of the studies were judged to have significant weaknesses with regard to sequence generation, allocation concealment, and/or blinding of outcome assessment. In none of the trials were the participants and personnel blinded. In four studies, the dropout rates at the post-intervention measurement exceeded 15%, potentially leading to distortions. All trials were considered to have a low risk of selective reporting. An overall Risk of Bias Graph is displayed in Fig. 2, while Fig. 3 provides a detailed summary of the Risk of Bias for each individual study.

### Meta-analyses of included studies

#### Perceived stress after intervention

**Fig. 4:**
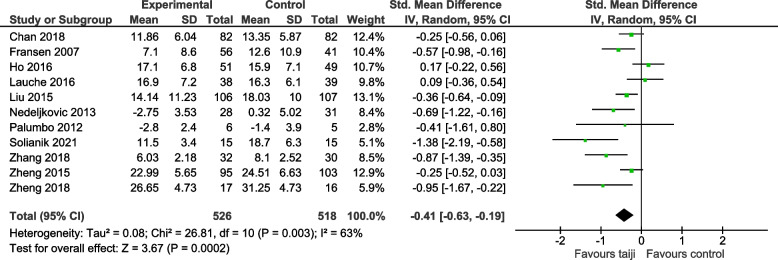
Forest plot for the primary outcome perceived stress (post-intervention)

**Fig. 5:**
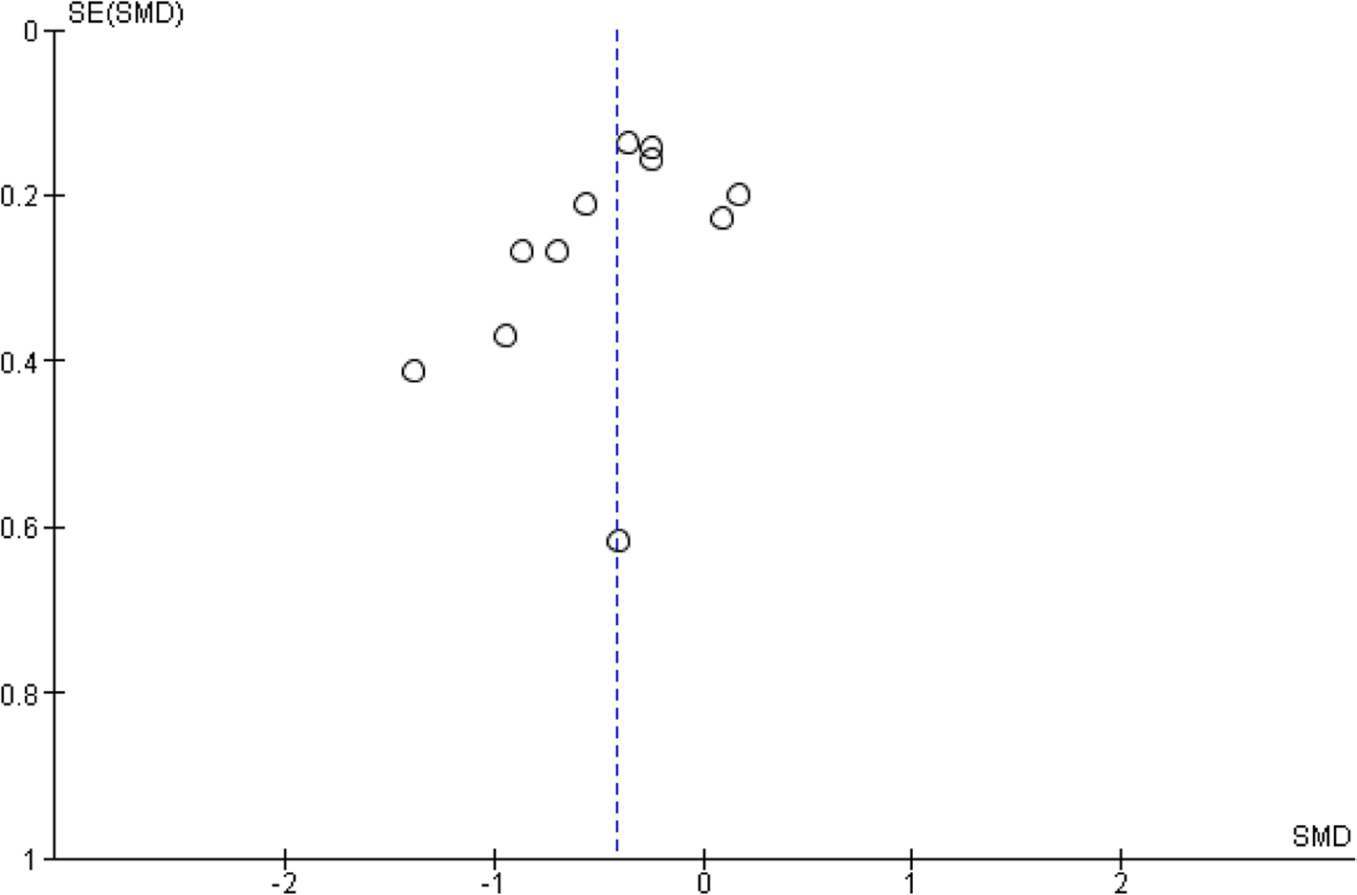
Funnel plot for perceived stress (post-intervention)

Figure 4 displays the forest plot for the primary outcome of perceived stress at the end of the intervention period. The pooled effect of taiji on perceived stress was significant (SMD -0.41, 95% CI -0.63 to -0.19; *p* < 0.001). There was moderate-to-large heterogeneity (I^2^ = 63%, *p* = 0.003). The Egger’s test for funnel plot asymmetry was not significant (asymmetry coefficient -2.05, *p* = 0.123), while the funnel plot suggested the presence of small study bias (Fig. 5).

We performed sensitivity analyses to assess potential influences of study quality characteristics on the SMDs for perceived stress. Specifically, we examined the impact of risk of bias concerning allocation concealment, blinding of study personnel, and incomplete outcome data. Given the possible presence of small study bias, we added a sensitivity analysis including only studies with sample sizes larger than 100 participants. Furthermore, we added a sensitivity analysis that included only studies with clearly predefined outcome measures (≤ 2 pre-specified primary outcomes, including how and when they were assessed). As indicated in Table 4, pooled effect sizes remained significant in all sensitivity analyses.

**Table 4:** Sensitivity analyses for the primary outcome perceived stress (post-intervention)

Sensitivity analysis	SMD (95% CI) random effects	I^2^ value
All studies (*n* = 11)	-0.41 (-0.53; -0.19)	63%
Low RoB – allocation concealment (*n* = 5)	-0.31 (-0.53; -0.09)	42%
Low RoB – blinding of outcome assessment (*n* = 5)	-0.43 (-0.76; -0.1)	75%
Low RoB – incomplete outcome data (*n* = 6)	-0.51 (-0.89; -0.13)	81%
Large sample size (≥ 100 participants) (*n* = 6)	-0.26 (-0.45; -0.06)	51%
Clearly predefined primary outcome measure (*n* = 4)	-0.54 (-1.01; -0.07)	70%

*Abbreviations: RoB* Risk of bias, *SMD* Standardized mean difference

Explorative regression analyses were performed to evaluate possible influences of sample characteristics on perceived stress including type of population, gender, age, intervention duration, and sample size (Table 5). Simple linear regressions revealed smaller treatment effects (*p* < 0.1) in studies with lower proportion of females (*p* = 0.024), in studies in patients (*p* = 0.055) and in larger studies (*p* < 0.001). In a next step, these variables were included in a multiple linear regression analysis to identify possible independent predictors for perceived stress. The overall model was statistically significant (*R*^2^ = 0.59, Q = 15.22, *p* = 0.002), with sample size (*p* = 0.013) turning out as the only independent predictor (Table 5).

**Table 5:** Explorative regression analyses for the primary outcome perceived stress (post-intervention)

Predictor	B	SE (95% CI)	Z	*P*-value	Beta
***Simple regression analyses***					
Age (years)	0.00	0.01 (-0.01; 0.03)	0.29	0.769	0.09
Females (%)	0.46	0.02 (0.06; 0.85)	2.25	0.024	0.59
Type of population (1 = others, 2 = patients)	0.3	0.15 (-0.01; 0.61)	1.92	0.055	0.38
Intervention duration (weeks)	0.02	0.03 (-0.04; 0.07)	0.61	0.545	0.18
Sample Size (0 = < 100; 1 = > = 100)	0.61	0.17 (0.28; 0.95)	3.60	< 0.001	0.73
***Multiple regression analyses***					
Intercept	-0.19	0.55 (-1.27; 0.88)	-0.35	0.724	0.00
Sample Size (0 = < 100; 1 = > = 100)	0.53	0.21 (0.11; 0.96)	2.50	0.013	0.63
Females (%)	-0.01	0.01 (-0.02; 0.0)	-1.34	0.182	-0.28
Type of population (1 = others, 2 = patients)	-0.01	0.17 (-0.33; 0.32)	-0.04	0.970	-0.01

#### Perceived stress at follow-up

**Fig. 6:**
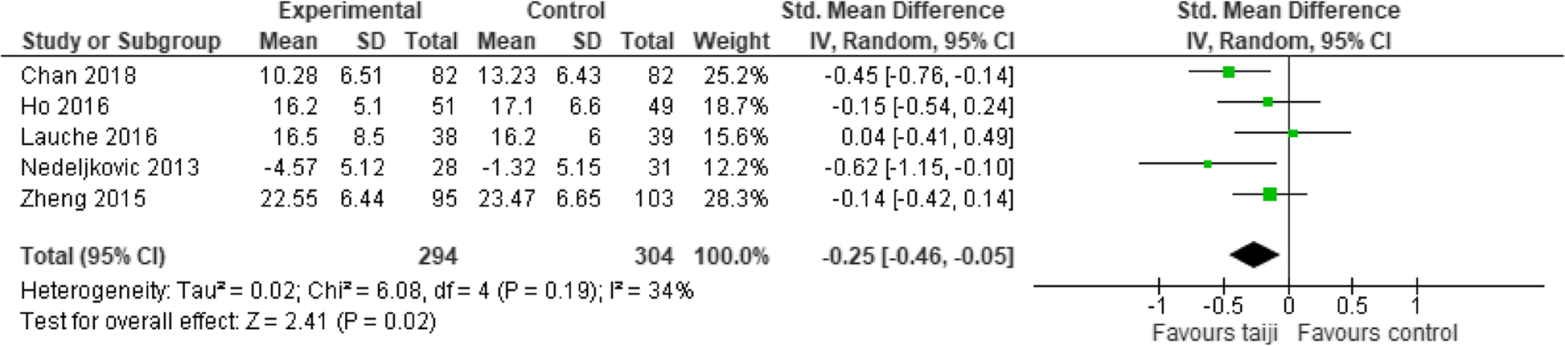
Forest plot for perceived stress at follow-up

Five studies provided a continuous outcome measure for perceived stress at follow-up (in median, 24 weeks after randomization) (Fig. 6). The SMD was smaller but still significant (-0.25, 95% CI -0.46 to -0.05; *p* = 0.02), and heterogeneity was low (I^2^ = 34%, *p* = 0.02).

#### Comparison with physical activity

**Fig. 7:**
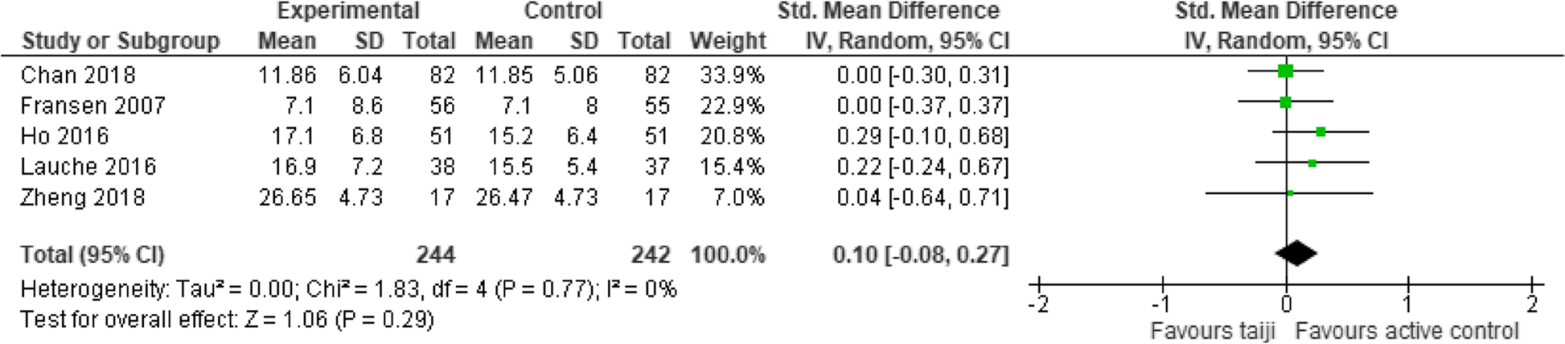
Forest plot for perceived stress in comparison to active control groups (post-intervention)

Additional analyses were performed to assess the effects of the interventions on perceived stress in comparison to active control groups (various forms of physical exercise). In the five studies that included such an additional group, the pooled SMD for perceived stress was not significant (SMD 0.10, 95% CI -0.08 to 0.27, *p* = 0.29; I^2^ = 0%) (Fig. 7).

#### Objective stress measures

**Fig. 8:**
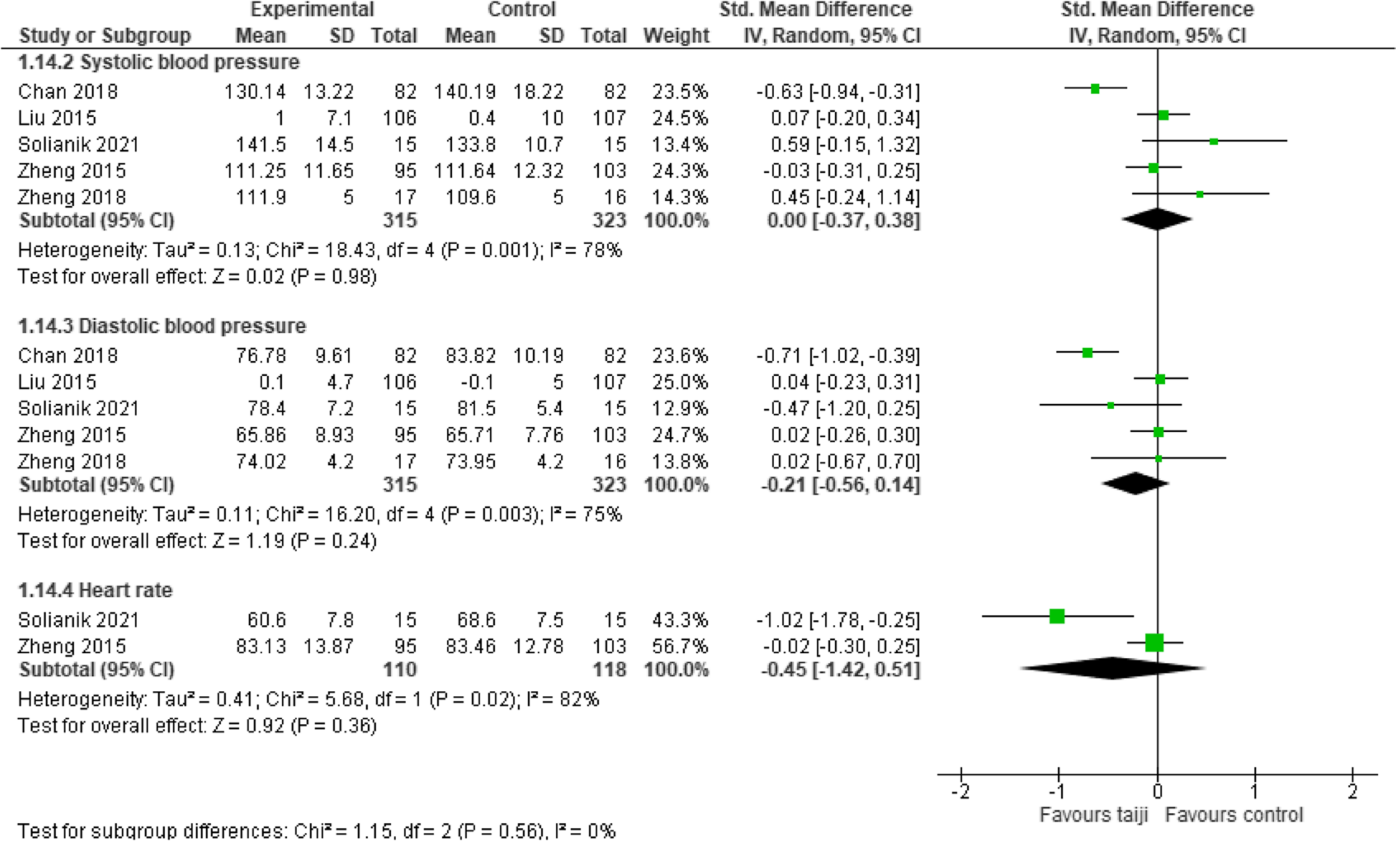
Forest plot for physiological stress measures (post-intervention)

Five RCTs provided at least one objective stress measure (5 trials systolic and diastolic BP, 2 trials heart rate). Neither systolic BP (SMD -0.0, 95% CI -0.37 to 0.38; *p* = 0.98; I^2^ = 78%, *p* < 0.001), nor diastolic BP (SMD -0.21, 95% CI -0.56 to 0.14; *p* = 0.24; I^2^ = 75%, *p* = 0.003), nor heart rate (SMD -0.45, 95% CI -1.42 to 0.51, *p* = 0.36; I^2^ = 82%, *p* < 0.02) showed a significant intervention effect in comparison to the NT control groups (Fig. 8).

#### Depression and anxiety

**Fig. 9:**
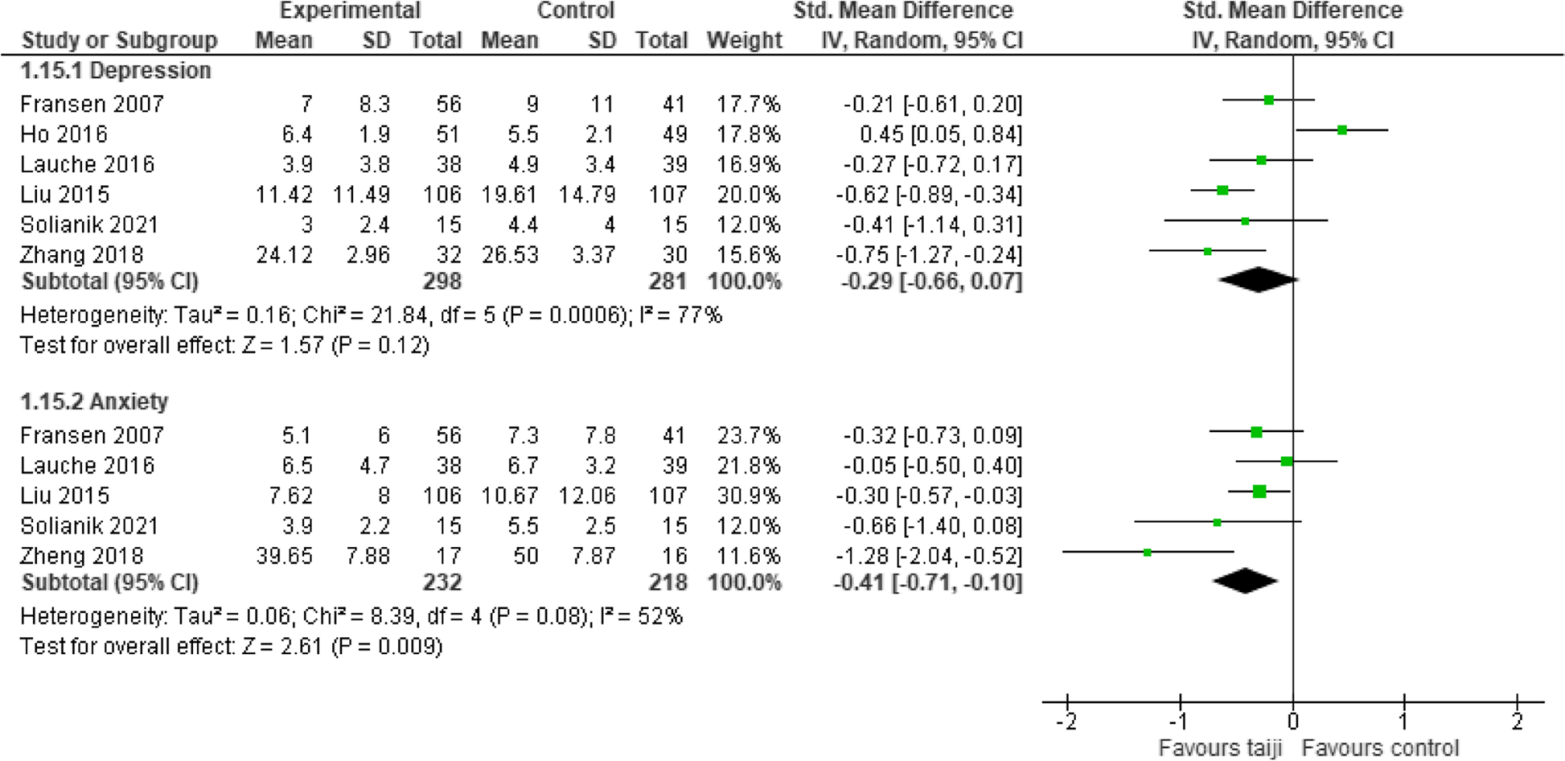
Forest plot for depression and anxiety

Six RCTs provided a measure for depression and five trials for anxiety. The pooled SMD for depression did not reach the level of significance (-0.29, 95% CI -0.66 to 0.07, *p* = 0.12), while heterogeneity was high (I^2^ = 77%, *p* < 0.001; Fig. 9). The pooled SMD for anxiety was significant (-0.41, 95% CI -0.71 to -0.1, *p* < 0.001), with moderate heterogeneity (I^2^ = 52%, *p* = 0.08) (Fig. 9).

#### Quality of life

**Fig. 10:**
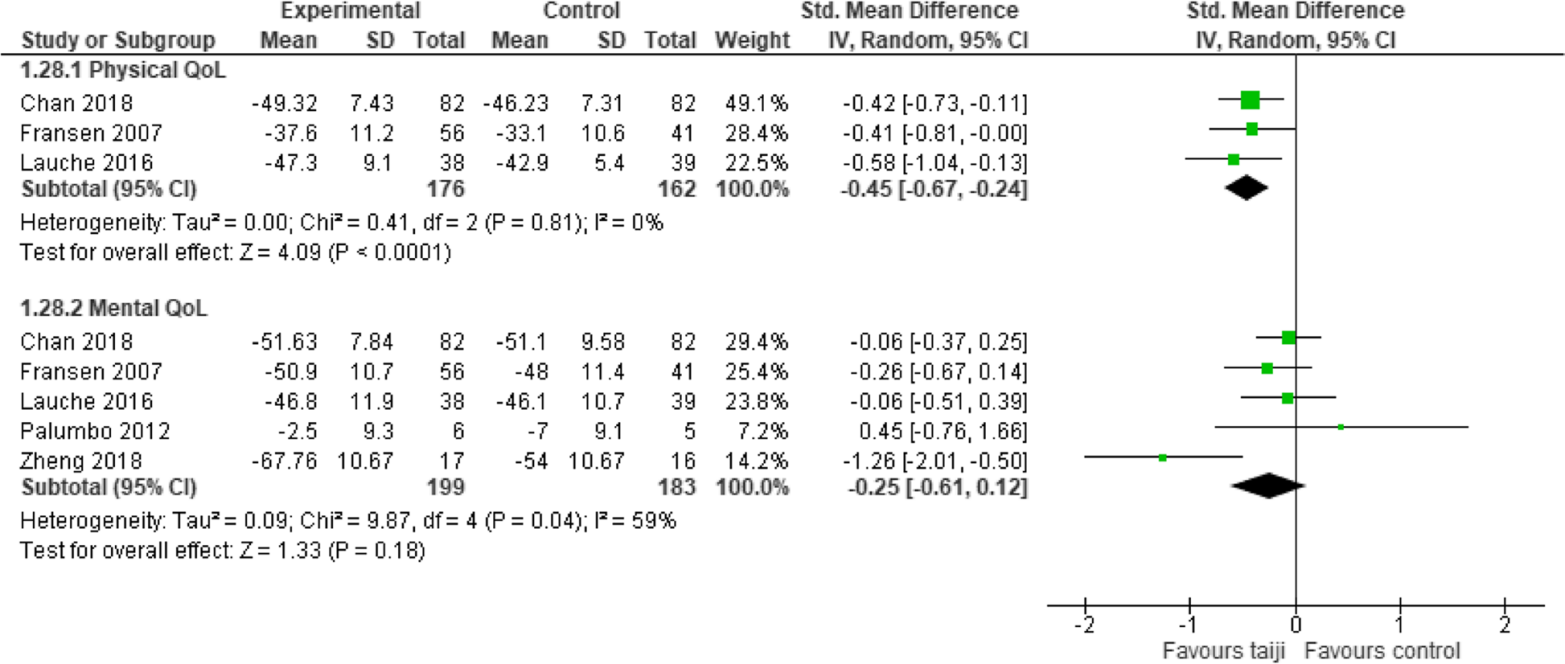
Forest plot for the secondary outcome quality of life (post-intervention)

Five studies reported a composite score for mental QoL and 3 studies for physical QoL. A significant intervention effect was revealed for physical QoL (SMD -0.45, 95% CI -0.67 to -0.24, *p* < 0.001; I^2^ = 0%), while the SMD for mental QoL was not significant (SMD -0.25, 95% CI -0.61 to 0.12, *p* = 0.18; I^2^ = 59%). (Fig. 10).

## Discussion

This systematic review examined and synthesized the evidence regarding taiji’s stress-relieving effects. Based on evidence from eleven RCTs included in the primary meta-analysis, taiji appears effective in reducing stress. The pooled SMD comparing taiji to no intervention showed a moderate effect size, with moderate-to-high between-study heterogeneity. The Funnel plot and the sensitivity analysis that excluded small studies both suggested the presence of small-study bias, with the latter yielding a reduced yet still significant SMD. Approximately half of the studies had notable weaknesses related to the definition of primary outcomes, allocation concealment, blinding of outcome assessment, and/or incomplete outcome data. Sensitivity analyses excluding these studies revealed reduced effect sizes in those with low risk regarding allocation concealment. The pooled SMD for perceived stress remained significant at 24-week follow-up. The stress-reducing effect of taiji in comparison to NT thus appears to be robust, enduring, and small-to-moderate in size.

Our exploratory simple regression analyses suggested smaller effects in patients and females; however, these influences were not confirmed by the multivariate regression analysis, in which only sample size emerged as an independent predictor. This confirms and extends prior research, which has demonstrated that taiji practice benefits mental health in diverse populations [24], comprising young and healthy subjects [1], adults [3], as well as patients [4, 5] and the elderly [25].

Five RCTs in this review included a third treatment group involving physical activity to control for body movement during taiji exercise. There was no evidence suggesting taiji had effects beyond those of regular physical activity. Interestingly, the health benefits of physical activity have been linked to its potential to dampen stress [26], which may explain the comparable effects on perceived stress.

Six studies reported results for one or more objective stress markers: five studies for systolic/diastolic BP and two for heart rate. However, the pooled SMD for neither measure indicated a significant effect of the intervention. This suggests that while taiji may influence perceived stress, it doesn't seem to impact the physiological stress response. In contrast, several reviews have highlighted reductions in BP resulting from taiji practice [27–29]. For instance, a recent meta-analysis found that regular taiji exercise could lead to clinically meaningful reductions in both SBP and DBP. This analysis also identified baseline BP as a major effect moderator, revealing larger BP reductions in hypertensive individuals and smaller reductions in those with normal BP [28]. Similarly, our findings showed the most pronounced positive effects of taiji on SBP and DBP in a study with hypertensive adults [23]. The lack of significant effects of taiji on BP in our review might therefore stem from the fact that most participants in the studies had normal BP. However, a preventive cardiovascular benefit of taiji practice might still be inferred, given taiji's regulatory effect on endothelial function and its potential role in maintaining healthy BP [27].

Stress is often accompanied by symptoms of depression and anxiety [30]. Our analyses indicate that practicing taiji had beneficial effects on anxiety. The SMD for depressive symptoms did not reach the level of significance. However, when we excluded one study that focused on patients with schizophrenia [21, 31] – a condition where depressive symptoms can overlap with negative symptoms [31], the SMD became significant (-0.47, 95% CI, -0.67 to -0.27, *p* < 0.001), and heterogeneity dropped from 77 to 13% (*p* = 0.33). More RCTs are needed to conclusively determine the impact of taiji on depressive symptoms.

Additionally, five studies presented a composite measure for physical and/or mental QoL. The results suggest small-to-moderate sized pooled effects for physical QoL, while the SMD for mental QoL was not significant. These findings align with a recent meta-analysis that evaluated taiji's effectiveness among older adults, both with and without chronic conditions. When QoL was analyzed only the physical but not the mental component showed significant improvement [32]. Other systematic reviews have highlighted taiji's beneficial impact on health-related QoL across various chronic conditions compared to NT, encompassing psychological factors [33–36]. However, the majority of these studies emphasized physical functioning and overall well-being. To draw definitive conclusions regarding taiji's specific impact on mental QoL, more research is needed that assesses both sub-dimensions.

Even though the present review clearly indicates that taiji is more effective than NT in reducing stress, it cannot answer the question of how much of the interventional effects of taiji are due to placebo effects, given the lack of placebo control groups. The effects of taiji could only be differentiated from other influences, such as natural history, regression to the mean, and unidentified co-interventions. For physicians, it is a professional ideal to use treatments only if their specific actions are proven [37]. However, one could argue that a self-help strategy like taiji is acceptable as long as it works better than NT. According to surveys in Switzerland and the US, the majority of patients (62%) would even accept medical interventions prescribed by a physician that are effective, even if a specific action has not been proven [38, 39]. Yet, the prerequisite for such a pragmatic approach is that the intervention is not harmful [40, 41], and certainly that necessary medical treatments are not overlooked. Since taiji is a non-invasive treatment and is typically used as an add-on treatment in patients with medical disorders, these prerequisites seem to be met. Nonetheless, taiji styles and forms should be adapted to a patient’s physical and mental state [42].

### Limitations

The present results are limited by their unblinded nature and the absence of placebo control groups. This could have led to positive expectations only in the treatment groups, thereby triggering placebo effects as well as a possible response bias. Other nonspecific influences, such as natural history and regression to the mean, could largely be ruled out by comparison to NT control groups. The methodological and reporting quality of the included RCTs varied widely across trials. However, sensitivity analyses revealed significant, albeit sometimes smaller, effect sizes for studies with higher methodological quality. One limitation of the results is the small number of trials, and especially the results for the secondary outcomes should be interpreted with caution. Another potential limitation arises from the inclusion of trials with perceived stress as an outcome, regardless of whether stress was the primary or secondary outcome in the respective trial, and irrespective of whether the study targeted patients or stressed yet healthy populations. Consequently, the primary stressors likely varied among studies, ranging from psychosocial stressors related to a high workload to those typically associated with chronic diseases (e.g., loss of work, financial problems, loneliness). Lastly, the typical duration of the interventions in this review comprised 24 weeks. The potential effects of taiji's long-term use on stress remain to be investigated.

## Conclusions

The scientific interest in the health-promoting effects of taiji has increased considerably during the past decades, and several new RCTs have been published in the past five years. Therefore, this systematic review aimed to evaluate the current evidence for the potential of this meditative movement intervention to reduce stress. Results indicate that, in comparison to untreated controls, taiji reduced perceived stress. The effect size was comparable in size to those associated with regular physical activity. Remarkably, taiji not only improved perceived stress but also anxiety and physical QoL. This broad effect on health and well-being is in accordance with the claim of integrative medicine to provide holistic care for the whole person rather than for single symptoms. However, more rigorously performed RCTs with sufficiently large sample sizes are needed to corroborate these findings.

## Data Availability

All data generated or analyzed during this study are included in this published article.

## Abbreviations

BP: Blood pressure
CI: Confidence interval
NT: No treatment
QoL: Quality of life
SMD: Standardized mean difference
WL: Waiting list
